# Hepatitis B: Knowledge, Vaccine Situation and Seroconversion of Dentistry Students of a Public University

**DOI:** 10.5812/hepatmon.13670

**Published:** 2013-10-05

**Authors:** Marina Sena Lopes da Silva Sacchetto, Simone Souza Lobão Veras Barros, Thaís de Alencar Araripe, Aryvelto Miranda Silva, Symonara Karina Medeiros Faustino, José Mário Nunes da Silva

**Affiliations:** 1 Department of Pathology and Clinical Dentistry (DPCO), Federal University of Piaui, Teresina, Brazil; 2 Department of Pharmacy, Differential Integral Faculty (FACID), Teresina, Brazil; 3 Department of Science and Health, Federal University of Piaui, Teresina, Brazil

**Keywords:** Immunization Schedule, Hepatitis B, Antibodies, Occupational Accidents

## Abstract

**Background:**

Viral hepatitis B (VHB) is an occupational risk for dentists. It is necessary that dental students start clinical practice immunized with the vaccine, response monitored and informed about the means of transmission of the disease. Rarely, there are studies, which evaluate concomitantly knowledge of these academics and their vaccine situation.

**Objectives:**

To evaluate the knowledge about Hepatitis B, the vaccine situation and the immunization status of dental students and to investigate the probable correlation between the status of immunization, vaccination membership and adherence to the test of seroconversion and associated factors.

**Patients and Methods:**

189 students from the dentistry course at the Federal University of Piaui (UFPI) who attended from the 3rd to 9th period were invited to participate in the research. Their knowledge about HBV, attitude regarding protection and their vaccine situation were assessed through a self-administered form. Antibodies against surface antigens of Hepatitis B virus (Anti-HBs) and against the antigens of the virus nucleous of Hepatitis B (Anti-HBc total) were measured qualitatively using the enzyme-linked immunosorbent assay (ELISA).

**Results:**

Of the 179 students surveyed, 58.1% knew about the degree of virulence of the Hepatitis B virus (HBV). As to the means of transmission, 98.3% considered blood transmission, 82.6% plates and cutlery, 15.6% cough and 12.3% vertical transmission. Most students (87.4%) knew that they should take 3 doses of the vaccine and 62.2% completed the immunization schedule. A minority of students (48.6%) knew the about the Anti-HBs test and 5.6% took the test. Among the students who reported having taken three doses of the vaccine, 12.5% were not seroconverted. There was no significant correlation between the variables.

**Conclusions:**

Dental academics were unsure about the means of infection and prevention against HBV. Many of them had not completed the immunization scheme and did not have the test of seroconversion. The serological analysis revealed unprotection, even after students completed the vaccination schedule.

## 1. Background

Viral hepatitis B is one of the most serious public health problems worldwide (1). There are more than two billion people infected in the world, of whom about 400 million are chronic carriers (2) and approximately 1 million people die annually because of the disease (3).

The transmission of the hepatitis B virus is by the parenteral route, and above all, it is transmitted sexually, being considered a sexually transmitted disease (4). Most people infected do not develop active liver disease, however, persistent infection can cause cirrhosis, liver failure or hepatocellular carcinoma (5). Liver transplant is the only hope for many patients with terminal liver diseases resulting from the HBV (6) and this represents a high cost to public health (5).

Surgeon-dentists are at increased risk of being infected by HBV (7, 8). The main methods of contamination include needle punctures or exposure to blood and other body fluids (9). These professionals are at up to ten times greater risk of acquiring Hepatitis B than an ordinary citizen (10). To prevent blood transmission of infection, it is recommended that health care professionals receive immunization against the disease (11) and use personal protective equipment (PPE) (12).

Vaccination represents the main instrument to prevent HBV infection (1, 13). Immunization should be carried out in three doses, with a month interval between the first and second dose and of six months between the first and the third dose, in order to stimulate the production of antibodies anti-HBs (4).

Although efforts have been made to vaccinate healthcare workers in Brazil, many do not vaccinate or do not complete the vaccination schedule (11). In addition, about 10% of these individuals do not produce sufficient anti-HBS after receiving the vaccination schedule (3, 14, 15) and therefore do not become protected. Thus, they must do the post-vaccination test until three months after the last dose of the vaccine (3).

The awareness of dentistry students about the measures that can prevent the transmission of Hepatitis B, is of great importance. It is necessary that they start clinical practice immunized with the vaccine, are response monitored and well informed about the possible transmission of viral infections in the dental office. Rarely, there have been studies that assess simultaneously the knowledge of dental students about Hepatitis B, their vaccination status and their immunization status. Furthermore, there are no reports of mandatory programs for vaccination together with the analysis of seroconversion in dental schools in Brazil.

## 2. Objectives

This study aims to evaluate knowledge about Hepatitis B, the vaccine situation and the status of academic dentists’ immunization, and to investigate the possible correlation between the status of immunization, vaccination membership and adherence to the test of seroconversion and associated factors.

## 3. Patients and Methods

### 3.1. Study Design

This was a cross-sectional observational study conducted on students of Dentistry of the UFPI.

### 3.2. Study Population

The sample was of censitary type, composed of students from the Dentistry course of the UFPI, who were from the 3rd to the 9th period, who accepted to participate in the research.

### 3.3. Data Collection

#### 3.3.1. Stages of the research

1st step: during the period from 14th to 27th January, 2013, a self-administered form, consisting of 29 closed questions and 3 open-ended questions, containing socio-economic-demographic information and about knowledge and practice of Hepatitis B control, was provided to the subjects. Before answering the form, the students signed an Informed Consent Form (ICF).

2nd Step: a campaign was conducted among academics of dentistry of UFPI from 28th January to 3rd February, 2013, emphasizing the importance of the study. In this campaign, students were invited to participate in the third stage of the study.

3rd Step: after signing a second ICF, the academics were submitted to blood collection for laboratory evaluation of serological markers. The examination was carried out from to 4th to 8th February 2013, by a nursing technique of the Central Laboratory of Piaui (LACEN/PI). 5 ml of blood was collected by venipuncture, using a syringe (10 ml) and disposable hypodermic needle, without anticoagulant.

### 3.4. Data Analysis

The blood was centrifuged to separate the serum used for research of antibodies. The serum samples were tested qualitatively for the serological markers Anti-HBs and total Anti-HBc. For this, we used the enzyme-linked immunosorbent assay test (ELISA) (BIOKIT, Barcelona, Spain), following the manufacturer's instructions.

#### 3.4.1. Anti-HBs

Certain parameters for the reading the results:

A-Positive: absorbance /cut-off ≥ 1, 0

B-Negative: absorbance /cut-off < 0, 9 

C-Doubtful: 0, 9 ≤ absorbance /cut-off < 1, 0

#### 3.4.2. Interpretation of the Results

Positive results for anti-HBs indicate immune response against HBV infection, immune response to the vaccine or the presence of passively acquired antibodies.

To determine if a student had positive results for anti-HBs, only due to prior contact with the virus and not the effect of the vaccine, an anti-HBc test using the ELISA test was used, following the manufacturer's instructions.

#### 3.4.3. Anti-HBc Total

Certain parameters for the reading the results:

A-Positive: absorbance/cut-off ≤ 1, 0 

B-Negative: absorbance/cut-off > 1, 1 

C-Doubtful: 1, 0 < absorbance/cut-off ≤ 1, 1.

#### 3.4.4. Interpretation of the Results

Anti-HBc positive result means that the patient has come into contact with the HBV. 

After laboratory analysis, the volunteers were classified into three profiles:

Profile 1- Immune by previous contact with the virus (Anti-HBc Total positive and Anti-HBs positive)

Profile 2- Immune by vaccination (Anti-HBc Total negative and Anti-HBs positive).

Profile 3- Individual susceptible (Anti-HBc total negative and Anti-HBs negative).

#### 3.4.5. Statistical Analysis

For analysis of the data, the Stata v.11.0 program was used (Stata Corporation, College Station, TX, USA). The univariate analysis was performed by descriptive statistics through frequencies and percentages. In bivariate analysis, to check the association between qualitative variables, the Pearson's Chi-square test (χ²) or FISHER test were employed as relevant and to measure the effect of exposure of variables (immunization status and knowledge about the degree of virulence of HBV) prevalence ratios (PR) were calculated and its respective confidence interval of 95% was considered statistically significant, p values ≤ 0.05.

### 3.5. Ethical Aspects

The research followed the standards contained in the Declaration of Helsinki governing research involving human beings and was approved by the Research Ethics Committee of the UFPI (08810412000005214).

## 4. Results

During the study period, 189 students were registered from the course of dentistry of the UFPI, from the 3rd to 9th period. From these candidates, 179 (94.7%) agreed to participate in the study, 2 were excluded from the study by participating directly in the research and 8 refused to participate. Of the 179 students, 56.4% were female and their average age was 21.9 (± 2.3).

During clinical care, 99.4% of the students wore gloves, mask and surgical cap, as protective measures. In Table 1 data on students ' knowledge about hepatitis B and the history of the participants and their families in relation to the disease are described.

**Table 1.** Students’ Knowledge about HVB and HVB Diagnosis in the Family Reported by the Research Participants (n = 179)

	No. (%)
**Do you know or have heard of Hepatitis B?**	
No	2 (1.1)
Yes	177 (98.9)
**Do you have or have you been diagnosed with hepatitis B?**	
No	176 (98.3)
Yes	03 (1.7)
**Hepatitis B in family history?**	
No	132 (73.7)
Yes	07 (3.9)
I don’t know	40 (22.3)
**Are there risks for people who live with the virus carrier?**	
No	39 (21.8)
Yes	120 (67.0)
I don’t know	20 (11.2)
**Is this disease curable?**	
No	62 (34.6)
Yes	82 (45.8)
I don’t know	35 (19.6)
**Is there any vaccine against this disease?**	
No	02 (1.1)
Yes	174 (97.2)
I don’t know	03 (1.7)
**Level of HBV virulence**	
Low	16 (8.9)
Medium	51 (28.5)
High	104, (58.1)
Ignored/No answer	08, (4.5)

**Figure 1.**
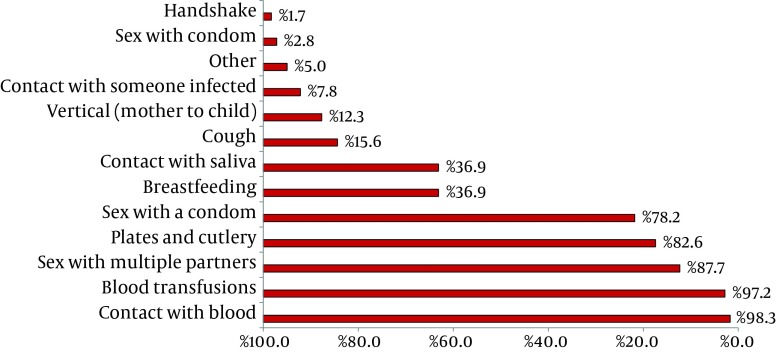
Main ways of Transmission Reported by the Research Participants (n = 179)

Students were asked about the ways of transmission of HBV. The result is summarized in Figure 1. 

The same process was done in relation to the ways of prevention. The alternatives commonly selected by the students were the use of PPE (88,2%), vaccination (92,7%) and the practice of safe sex (62,4%) and consumption of boiled water or treated (11,8%).

The responses of the questionnaires relating to questions about the vaccine situation and knowledge of the process of vaccination against HVB are summarized in Table 2. 

**Table 2.** Data Concerning Knowledge About Vaccination Against Hepatitis B, the Vaccine Situation and Anti-HBs test Reported by the Research Participants

	No. (%)
**Are you vaccinated against Hepatitis B? (n = 179)**	
No	17 (9.5)
Yes	143 (79.9)
I don’t know	19 (10.6)
**If not why? (n = 36)**	
No interest	02 (5.6)
Forgetfulness	18 (50.0)
Other	06 (16.7)
No answer	10 (27.8)
**Took all doses? (n = 143)**	
No	33 (23.1)
Yes	98 (68.5)
I don’t know	12 (8.4)
**How many doses did you get? (n = 143)**	
1 dose	02 (1.4)
2 doses	07 (4.9)
3 doses	89 (62.2)
No answer/Ignored	45 (31.5)
**How many doses should be taken? (n = 179)**	
1 dose	04 (2.8)
2 doses	04 (2.8)
3 doses	125 (87.4)
4 doses	03 (2.1)
No answer/Ignored	43 (4.9)
**How long has it been since the completion of the vaccination schedule? (n = 143)**	
≤ 6 months	13 (9.1)
> 6 months	85 (59.4)
No answer/Ignored	45 (31.5)
**Did you Know or have heard about Anti-HBs test? (n = 179)**	
No	92 (51.4)
Yes	87 (48.6)
**If Yes, where did you obtain this information (n = 87)**	
University classes	70 (80.5)
In articles, books etc.	18 (20.7)
Internet	11 (12.6)
Other	01 (1.2)
Specialized establishments	07 (8.0)
**Have you had the Anti-HBs test ?(n = 179)**	
No	169 (94.4)
Yes	10 (5.6)
If not why? (n = 169)	
I don’t know the test	81 (47.9)
Forgetfulness	33 (19.5)
I don’t have no interest	02 (1.2)
Other	45 (26.6)
No answer/Ignored	08 (4.7)
**If Yes, how long after the last dose of vaccination? (n = 10)**	
≥ 6 months	02 (20.0)
< 6 months	08 (80.0)

### 4.1. Laboratorial Step

Of the 179 students who responded to the form, 159 (88.82%) participated in the blood collection. This sample loss was attributed mostly to the fear of the individuals from blood withdrawal. The loss was analysed and it was found by the Pearson’s Chi-square test that there was no significant difference between the variables: gender (P = 0, 539), age group (P = 0, 262), period of the course (P = 0, 577) and thus clinical practice was initiated (P = 0, 741) with the existing losses.

As for the serological analysis of anti-HBs, 126 (79.2%) of the 159 students assessed were positive and 33 (20.8%) were negative.

In the analysis of total anti-HBc, it was found that only 1 academic was positive, however, this student was anti-HBs negative. Thus the profile of the academics regarding protection against HBV was thus distributed:

Profile 1 (immune by previous contact with the virus): 0,6% 

Profile 2 (Immune by vaccination): 79.2% 

Profile 3 (Individual susceptible): 20.2%

### 4.2. Bivariate Analysis

There was no statistically significant association between the Anti-HBs and the variables analyzed (Table 3). 

**Table 3.** Relationship Between Gender, Age, Number of Doses and Time of Completion of the Vaccination Scheme with the Immunization Status

Variables	Anti-Hbs		Total, No. (%)	RP ^a^(CI ^a^ 95%)	P value ^b^
	Positive No. (%)	Negative No. (%)			
**Gender**					0.888
Female	67 (78.8)	18 (21.2)	85 (100.0)	Ref ^a^	
Male	59 (79.7)	15 (20.3)	74 100.0	0.96 (0.48-1.90)	
**Age, y**					0,320
18 - 20	31 (81.6)	07 (18.4)	38 100.0	ref.	
21 - 23	75 (81.5)	17 (18.5)	92 100.0	0.77 (0.39-1.53)	
≥ 24	20 (69.0)	09 (31.0)	29 100.0	1.68 (0.78-3.61)	
**Number of doses **					0.122 ^c^
1	01 (50.0)	01 (50.0)	02 100.0	ref.	
2	05 (71.4)	02 (28.6)	07 100.0	2.13 (0.47-9.61)	
3	70 (87.5)	10 (12.5)	80 100.0	0.37 (0.10-1.36)	
**Time of vaccination conclusion, mo**					0.444 ^c^
≤ 6	11 (91.7)	01 (8.3)	12 100.0	ref.	
> 6	65 (84.4)	12 (15.6)	77 100.0	1.87 (0.24-14.38)	

^a^ Abbreviations: CI, confidence interval; RP, ratio of prevalence; ref, reference category

^b^ Chi-square Test χ²

^c^ FISHER

There was no statistically significant result regarding the relationship between gender (P = 0, 542), period of the course (P = 0, 616), history of hepatitis B in the family, history of occupational accident (P = 0, 211) and the knowledge of immunization status. Also, there was no meaningful result on the relationship between gender (P = 0,818), period of the course (P = 0, 492), history of hepatitis B in the family (P = 0, 615), history of occupational accident (P = 0, 981) and the account of having completed the vaccination scheme.

## 5. Discussion

Dental students should know the ways of transmission and prevention against HBV, since the proper attitude against the disease is the primary way, which is to prevent its spread (16, 17). The results of this study indicate that the students of the dentistry course of UFPI who attend the 3rd to 9th periods, parts of the course in which there is clinical practice, do not demonstrate safety regarding transmission of HVB.

In this study, few scholars considered vertical transmission as a form of contagion, and most of them pointed to the common use of plates and cutlery as the transmission medium. These results suggest that students confuse the contamination means of various types of hepatitis. In addition, some surveyed participants thought that coughing transmits Hepatitis B, and many of them demonstrated that they did not know HBV virus as highly virulent. This study is consistent with a survey conducted in Iran (16) and India (17). Already, a study of dental students in Taiwan (18) revealed that they are knowledgeable about the disease and its means of transmission.

It was possible to observe a contradictory behavior between the academics, since most cited the vaccine as a way of prevention, but little more than half of the students reported having completed the vaccination schedule. The low adhesion to the vaccine program is not justified, considering that, in Brazil, vaccination is free of charge and released to health care students (19). These results resemble those pointed out by Sing A et al. (2010) (19) and Acosta-Gió AE et al. (2008) (20), but differ from other results (7, 9, 18, 21-23) in which the percentage of completion of the vaccination scheme varies from 73, 8% to 97%.

Seroconversion is the appearance of antibodies against HBV in the blood in an amount sufficient to ensure immunity (11, 24-28). In this study, less than half of the students were aware of the seroconversion test and only 5.6% performed the test. This result does not resemble that reported by other researches (7, 9, 22) where the percentage of seroconversion analysis ranged from 14.8% to 47.88%.

The attitude of the dentist towards completion of the immunization schedule and to check if he is immunized may be related to other characteristics. In the survey among dentists in Belo Horizonte (Brazil), women and professionals who had HVB in their family history, completed the vaccination schedule most frequently (22). In the present study, these variables did not influence the behavior of students, perhaps for the small amount of sample.

In the present study, there was no significant relationship between the course period and the knowledge of immunization status and/or the full vaccination, revealing the need for continuing education, a result also found in a study with Iranian academics of dentistry (9). However, these results do not resemble those of a survey with students of Dentistry of Croatia, in which it was evidenced that the attitudes and knowledge regarding HBV improve in the course of graduation (29).

In this research, of the 159 academics who accepted to participate in the laboratory step, only 1 had a positive outcome for total Anti-HBc and this student was negative for Anti-HBs, probably due to the decreased concentration of Anti-HBs to levels undetectable by serological tests over the years (4). This result is similar to those of a survey conducted at the University of Bahia (Brazil) (11) and a study conducted at the Mato Grosso (Brazil) (30).

Regarding the result of Anti-HBs, 79.2% of the students were considered immune by vaccination response, and 20.2% were considered susceptible. However, when the analysis was performed, only among students who reported having completed the vaccination, the percentage of people unprotected decreased to 12.5%, showing the importance of completing the scheme. In the literature, the percentage of healthcare professionals who remain unprotected after 3 doses of vaccine, ranged from 5.85% to 20.9% (7, 24, 30, 31).

This difference in seroprotection is probably due to several factors that supposedly can influence the concentration of anti-HBs. Studies have shown that age (7, 9, 32) and the time of vaccination (7, 9, 13, 24, 33) negatively influence the amount of antibodies after completion of the immunization schedule, the number of doses taken increases the concentration of anti-HBs (32) and gender has no influence on seroconversion (7, 9, 30). In this study none of the variables analyzed, influence the concentration of anti-HBs.

The time of the vaccine protection against Hepatitis B continues to be widely discussed (34). This research has some limitations such as the fact that the sample size was small and there was a lack of confirmation of the reports, because students did not have a vaccination card.

In conclusion, the results of this study demonstrated that dental students were unsure about the ways of infection and prevention of Hepatitis B, and a large part did not complete the vaccination before starting clinical practice and a small percentage performed this test. Serological analysis confirmed the literature and revealed unprotected pupils, even after completion of the vaccination. It is suggested that during the graduation, education campaigns on the disease must take place and the delivery of the Anti-HBs test results must be a pre-requisite for the onset of clinical practice.
